# Clomiphene citrate reduces premature LH surge in obese women during controlled ovarian stimulation: a retrospective cohort study

**DOI:** 10.3389/fendo.2025.1512821

**Published:** 2025-03-21

**Authors:** Yuqi Zeng, Yuting Huang, Yali Liu, Xi Shen, Yunhan Nie, Li Wang, Yanping Kuang

**Affiliations:** Department of Assisted Reproduction, Shanghai Ninth People’s Hospital, Shanghai Jiao Tong University School of Medicine, Shanghai, China

**Keywords:** obesity, clomiphene citrate (CC), luteinizing hormone (LH), *in vitro* fertilization (IVF), assisted reproductive technology (ART)

## Abstract

**Background:**

Clomiphene citrate effectively suppressed the negative feedback of estrogen on the hypothalamus and induces premature luteinizing hormone (LH) surge during controlled ovulation stimulation, while obese women often have impaired hypothalamic-pituitary function. This study aimed to investigate whether the utilization of clomiphene citrate for controlled ovulation stimulation in obese women can effectively decrease the likelihood of premature LH surge.

**Methods:**

A retrospective study was conducted on women under the age of 38 with normal menstrual cycles and ovarian reserve who underwent controlled ovulation stimulation (COH) using clomiphene citrate. The participants were categorized by the Asian body mass index (BMI) classification. The dynamic reproductive endocrinological profiles during COH, especially the probability of serum LH concentration exceeding 10 IU/L, as well as the outcomes related to the embryo development and pregnancy, were compared among three BMI groups.

**Results:**

The basal levels of LH exhibited a significant reduction in overweight and obese women (p<0.001). Additionally, there was a significant decrease in the incidence of LH concentration exceeding 10 IU/L during controlled ovulation stimulation among overweight and obese women (7.19% vs 3.62% vs2.27%, p<0.05). Moreover, there were no significant differences observed among the three BMI groups regarding embryo transfer numbers, implantation, pregnancy or live birth rates.

**Conclusions:**

In obese women, clomiphene citrate effectively controlled LH levels, resulting a low prevalence of premature LH surge compared to patients with normal weight. This evidence contributes to a safer and more effective treatment for infertility in obese individuals.

## Introduction

Clomiphene citrate (CC), a selective estrogen receptor antagonist (1), effectively suppresses the negative feedback of estrogen on the hypothalamus, thereby stimulating pituitary gonadotrophins and enhancing ovarian sensitivity (2). CC’s ability to suppress estrogen’s negative feedback results in an elevation of LH levels during ovarian stimulation, thereby increasing the occurrence of premature LH surges. In 1985, Messinis et al. observed that all 12 patients exhibited premature LH surges on days 7-9 of human menopausal gonadotropin (HMG) administration when using a combination of 150 mg CC and 225 IU HMG daily for ovarian stimulation (3). Similarly, spontaneous LH surges were observed in 97 out of 314 cycles of oocyte retrieval with CC, indicating an incidence of 30.89% (4). Terakawa et al. demonstrated that pretreatment with CC followed by estradiol (E2) administration could induce LH surge in ovariectomized rats, while E2 alone was ineffective.

Obese patients exhibit a general decrease in hypothalamic-pituitary function, affecting not only the gonadal axis but also the growth hormone axis, thyroid axis, and so on (5–7). The levels of luteinizing hormone (LH) and follicle-stimulating hormone (FSH) are diminished in women with obesity, along with a decrease in the production of sex steroids, attributed to a relative decline in pituitary function (8). Additionally, obesity impairs ovarian responsiveness to gonadotropin stimulation, resulting in the requirement for increased gonadotropin dosage and duration (9, 10). The impaired hypothalamic-pituitary function in obese women may influence their response to CC, a factor that has not been extensively explored.

Considering CC’s ability to enhance LH levels and the observed low LH levels in patients with obesity, we hypothesize that clomiphene citrate compensates for diminished hypothalamic-pituitary function in obese women, and leading to reduced premature LH surge. Therefore, reproductive endocrinological profiles, specifically dynamic LH levels, as well as oocyte, embryo, and pregnancy outcomes were compared between patients with obesity and normal weight patients undergoing COH with CC.

## Materials and methods

### Ethics approval

This retrospective study was approved by the ethics committee of Shanghai Ninth People’s Hospital, Shanghai Jiao Tong University School of Medicine (SH9H-2024-T134-1). Due to the retrospective nature, no identifiable data were available to the researchers.

### Study setting and patients

**Figure 1:**
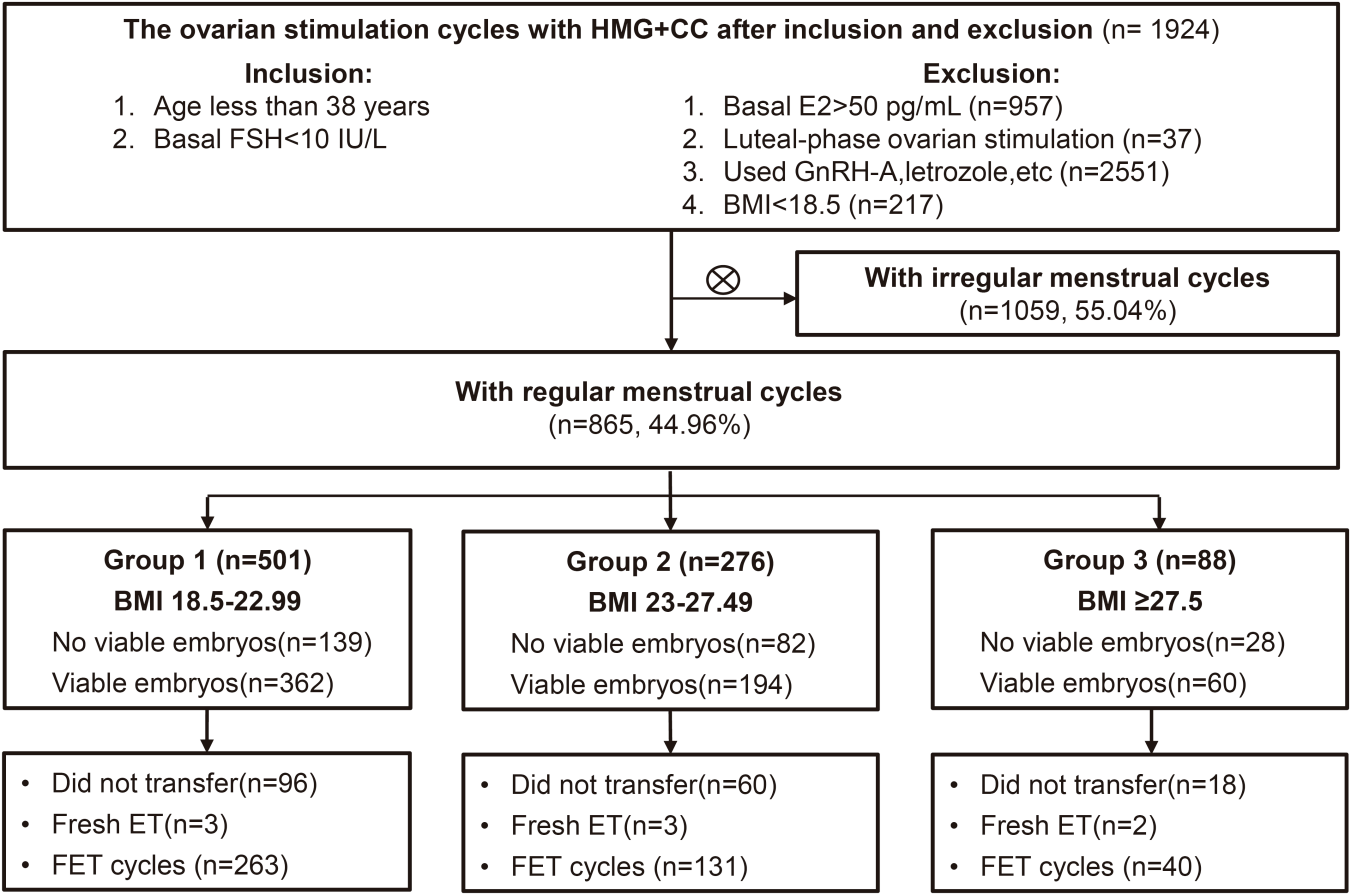
Flow chart illustrating the study population, encompassing the inclusion and exclusion criteria. IVF, *in vitro* fertilization; ICSI, intracytoplasmic sperm injection treatment; FSH, follicle-stimulating hormone; BMI, body mass index; ET, embryo transfer. FET, frozen embryo transfer.

This retrospective cohort study was conducted at the Department of Assisted Reproduction of the Ninth People’s Hospital affiliated to Shanghai Jiao Tong University School of Medicine. From January 2010 through September 2023, women undergoing IVF/ICSI regimens for the treatment of infertility were recruited as following criteria: [1] age less than 38 years, [2] regular menstrual cycles over the previous three months (24-38 days in duration (11)), [3] normal ovarian reserve (FSH no more than 10 IU/L on menstrual cycle day 2-4), [4] the controlled ovarian stimulation was carried out using the HMG and clomiphene citrate regimen. The exclusion criteria were: [1] basal E2>50 pg/mL, [2] luteal-phase ovarian stimulation, [3] patients who have used GnRH antagonists, letrozole, etc. to interfere with LH levels, [4] body mass index (BMI) less than 18.5 since the endocrine characteristics of underweight subjects may differ significantly (12, 13) and fall outside the scope of our study’s focus. The final enrolled patients were divided into three groups according to the modified classification of BMI for the Asian population (14): those with a BMI between 18.5–22.99 kg/m^2^ were considered to have a normal BMI (group 1), those with a BMI of 23–27.49kg/m^2^ were classified as overweight (group 2), and those with a BMI over 27.5 kg/m^2^ were categorized as having obesity (group 3). The flow diagram is presented in **Figure 1**.

In the normal weight group, individuals under age of 30 accounted for 24.8% (n=124), those aged 30-34 made up 48.1% (n=241), and individuals aged 35-37 constituted 27.1% (n=136). In the overweight group, individuals under age of 30 represented 25% (n=69), those aged 30-34 comprised 41.3% (n=114), and individuals aged 35-37 accounted for 33.7% (n=93). In the obese group, individuals under age of 30 made up 17.0% (n=15), those aged 30-34 represented 63.6% (n=56), and individuals aged 35-37 constituted 19.3% (n=17).

### Controlled ovarian hyperstimulation, oocyte retrieval and *in vitro* fertilization/intracytoplasmic sperm injection

The COH regimen used in all enrolled patients was the HMG+CC regimen. Patients were administered 150 IU/d of HMG (Anhui Fengyuan Pharmaceutical Co., China) and 50 mg/d of CC (Fertilan; Codal-Synto Ltd., France) from menstrual cycle day 3. Transvaginal ultrasonography was performed after 3-5 days to record the size and number of developing follicles. The concentrations of serum FSH, LH, E2 and progesterone were measured at the same time. HMG doses were adjusted based on the ovarian response, with a daily range of 150-300 IU. Oocyte maturation was triggered by triptorelin (0.1 mg; Decapeptyl, Ferring Pharmaceuticals, Germany) and HCG (2000 IU; Lizhu Pharmaceutical Trading Co., China) when at least three follicles reached diameters of 18 mm or more. Oocyte retrieval was conducted 34 to 38 hours after trigger, and all follicles with diameters of more than 10 mm were aspirated.

Fertilization was performed *in vitro* using either IVF or ICSI following oocyte retrieval, based on the semen parameters and previous fertilization outcomes. Following IVF/ICSI, embryos were evaluated for the number or regularity of blastomeres and the degree of fragmentation based on the Cummins’ criteria (15). The high-quality cleavage-stage embryos (grade 1 and grade 2, with at least six cells) were frozen via vitrification (16) on the 3rd day after oocyte retrieval. The surplus good-quality embryos (more than six) and non-top-quality embryos underwent blastocyst culture. Blastocysts with good morphology were frozen either on day 5 or day 6 (17).

The endometrial preparation schemes for frozen embryo transfer (FET) cycles included natural cycle, hormone replacement cycle, letrozole mild stimulation cycle and HMG late stimulation cycle, as previously described in our previous work (18, 19). Each patient received no more than two embryos per cycle and progestin (P) supplement was continued until eight weeks of gestation if pregnancy was achieved.

### Hormone measurement

The hormone levels were measured with chemiluminescence (Abbott Biological B.V. Netherlands) at the laboratory of Shanghai Ninth People’s Hospital, which is a routine and reliable examination method. The lower limits of sensitivity were as follows: FSH, 0.06 IU/L; LH, 0.09 IU/L; E2, 10 pg/mL; and P, 0.1 ng/mL. The upper limit of E2 concentration was 5,000 pg/mL. If serum E2 on the trigger day or the day after was higher than the upper limit, it was documented as 5000 pg/ml.

### Statistical analysis

The primary outcome measure for this study was the dynamic reproductive endocrinological profiles during COH, especially the probability of serum LH concentration exceeding 10 IU/L, which was considered indicative of premature LH surge (20–22). The secondary outcomes were the quantitative changes in the number of follicles to oocytes retrieved, average estrogen concentration per follicle, duration and dosage of HMG administration, viable embryos and clinical pregnancy outcomes from the first FET cycle following oocyte retrieval. The implantation rate was defined as the number of gestational sacs divided by the number of embryos transferred. Clinical pregnancy was defined as a pregnancy diagnosed by ultrasound evidence of one or more gestational sacs or clear clinical signs of pregnancy. The miscarriage rate is the total number of induced and spontaneous abortions divided by the number of clinical pregnancies.

Continuous variables were presented as mean ± standard deviation and tested using one-way ANOVA. Categorical variables were expressed as proportions (%) and tested using chi-square test or Fisher’s test. *Post hoc* analyses were performed using the Bonferroni test, and P < 0.05 was considered statistically significant. All statistical analyses were conducted using SPSS (version 25).

## Results

### Characteristics of women with normal and high BMI when using clomiphene citrate

After applying the inclusion and exclusion criteria, the study enrolled a total of 875 women, categorized as follows: 501 women with a normal BMI were placed in group 1, 276 women with a BMI indicating overweight were placed in group 2, and 88 women with a BMI in the obesity category were identified as group 3. In the case of subsequent embryo transfer, 249 women did not receive viable embryos, 8 women underwent fresh embryo transfers, and 177 women did not undergo embryo transfer, leaving a total of 434 cycles for the first FET of this oocyte retrieval (**Figure 1**). The baseline demographic characteristics of the three groups are shown in **Table 1**. BMI and infertility duration were significantly higher in group 3 patients than those in group 2 and group 1. There were no significant differences in age, primary infertility and causes of infertility between groups.

**Table 1:** Baseline characteristics and IVF/ICSI outcomes of women with clomiphene citrate by BMI category (kg/m^2^).

	Group 1 (BMI 18.5-22.99)	Group 2 (BMI 23-27.49)	Group 3 (BMI≥27.5)	*P* value
**Number of women**	501 (57.92%)	276 (31.91%)	88 (10.17%)	
**Age (year)**	31.90±3.25	32.21±3.60	31.95±2.82	0.444
**Body mass index (kg/m^2^)**	20.85±1.21^a^	24.76±1.25^b^	30.24±2.44^c^	**<0.001**
**Infertility duration (year)**	3.09±2.81^a^	3.60±3.14^b^	3.59±2.71^ab^	**0.040**
**Primary infertility (n, %)**	300 (59.88%)	165 (59.78%)	54 (61.36%)	0.962
**Causes of infertility (n, %)**				0.368
Tubal factor	253 (50.50%)	135 (48.91%)	44 (50.00%)	
Male factor	42 (8.38%)	30 (10.87%)	6 (6.82%)	
Endometriosis	25 (4.99%)	8 (2.90%)	1 (1.14%)	
PCOS	1 (0.20%)	2 (0.72%)	0 (0.00%)	
Combined factor	109 (21.76%)	57 (20.65%)	18 (20.45%)	
Unexplained factor	71 (14.17%)	44 (15.94%)	19 (21.59%)	
**hMG duration (d)**	6.96±2.71^a^	7.46±3.18^b^	8.45±3.21^c^	**<0.001**
**hMG dose (IU)**	1216±659^a^	1406±853^b^	1837±955^c^	**<0.001**
**Antral follicle count (n)**	7.54±5.29^a^	7.93±6.88^a^	9.51±7.27^b^	**0.019**
**Follicles > 10 mm on hCG day (n)**	7.12±5.04	6.54±4.96	6.74±4.78	0.282
**Follicles> 14 mm on hCG day (n)**	5.12±3.96	4.58±3.20	4.68±3.40	0.125
**Oocytes retrieved (n)**	5.23±4.36	4.50±3.68	4.72±4.20	0.055
**Mature oocytes (n)**	4.16±3.42	3.74±3.22	4.05±3.84	0.257
**Fertilized oocytes (n)**	3.73±3.18	3.28±2.78	3.73±3.56	0.133
**Cleaved embryos (n)**	3.25±2.87	2.91±2.61	3.25±3.12	0.244
**Viable embryos (n)**	1.62±1.63	1.53±1.59	1.75±1.93	0.523
**Top-quality embryos (n)**	1.29±1.45	1.28±1.49	1.52±1.81	0.376
**Oocyte retrieval rate (%)**	65.90±27.29	64.90±26.04	66.32±28.06	0.857
**Mature oocyte rate (%)**	80.85±27.15	82.42±28.34	82.96±29.05	0.663
**Fertilization rate (%)**	85.49±28.39	84.93±30.14	86.89±28.11	0.858
**Cleavage rate (%)**	80.72±32.75	81.09±34.08	79.04±34.41	0.880
**The rate of no embryo (n, %)**	139 (27.74%)	82 (29.71%)	28 (31.82%)	0.679

For continuous data, a one-way ANOVA analysis was performed; for proportions, the chi-square test was utilized. *Post hoc* analyses were performed with the Bonferroni test. Different superscript letters indicate significant differences between groups. BMI, body mass index; PCOS, polycystic ovary syndrome; hMG, human menopausal gonadotropin; hCG, human chorionic gonadotropin. P < 0.05 was considered statistically significant.

Bolded words in the table denote the titles of the indicators, while bolded values signify statistical significance.

### Embryo outcomes of women with normal and high BMI when using clomiphene citrate

**Figure 2:**
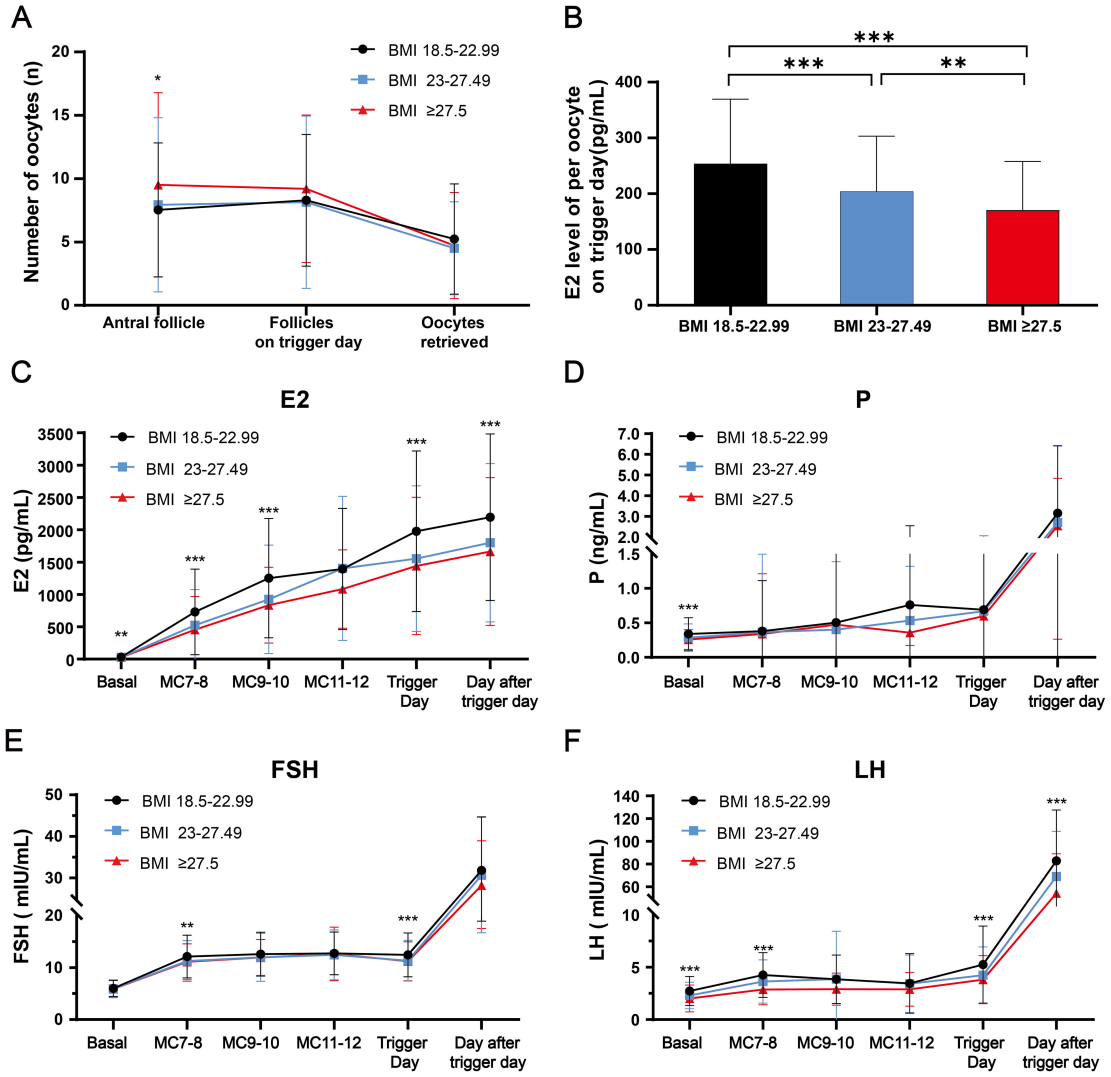
The dynamic follicular and reproductive endocrine changes during controlled ovarian hyperstimulation with clomiphene citrate in women with normal and high BMI were investigated. **(A)** The dynamic changes from antral follicle, follicles on trigger day, to the retrieved oocytes among three BMI groups were analyzed. **(B)** The average estrogen concentration per follicle on trigger day was compared among three BMI groups. **(C–F)** The dynamic changes of E2, P, FSH and LH levels during the controlled ovarian hyperstimulation with clomiphene citrate in women with normal and high BMI was examined. Comparisons of differences between groups were conducted using one-way ANOVA followed by *post hoc* analyses performed with the Bonferroni test. *: p<0.05; **: p<0.01; ***: p<0.001. BMI, body mass index; P, progesterone; FSH, follicle-stimulating hormone; LH, luteinizing hormone.

The cycle characteristics of COH in different BMI groups are shown in **Table 1**. The groups of higher body mass index had a longer stimulation duration and consumed more HMG. There was no significant difference between the different BMI groups in mature oocytes, fertilized oocytes, cleaved embryos, viable embryos, top-quality embryos, oocyte retrieval rate, mature oocyte rate, fertilization rate, cleavage rate and no viable embryo rate. We further explored the quantitative changes from antral follicle to total follicles on trigger day to oocytes retrieved, and the average estrogen concentration per follicle on trigger day in **Figures 2A, B**. Group 3 (people with obesity) and group 2 (people who were overweight) had higher antral follicle counts than group 1 (people with normal weight). There was no difference in total follicles on trigger day between groups, but the number of oocytes retrieved tended to be lower in group 3 and group 2 compared to the normal weight group (P=0.055). The E2 level per oocyte on trigger day was calculated to reflect the ability of each follicle to produce estrogen, and group 3 and group 2 were found to have lower E2 levels per oocyte on trigger day than group 1, representing less estrogen produced.

### Dynamic serum levels of FSH, E2, and P in women with normal and high BMI when using clomiphene citrate

The dynamic changes in serum concentrations of E2, P and FSH related to reproductive endocrine function are presented in **Figures 2C-E**. E2 gradually increased, accompanied by the development of multiple follicles during COH, with group 2 and group 3 having lower estrogen levels than group 1 at most times (P < 0.01). Group 1 had higher basal serum P levels compared to groups 2 and 3 (P < 0.001). However, there was no significant difference in the gradual rise during COH and rapid rise after triggering among all three groups for progesterone. As for FSH, group 2 and group 3 had mildly lower FSH than group 1 at early-follicular phase and trigger day (P < 0.01), with no difference at other times.

### Dynamic serum LH levels in women with normal and high BMI when using clomiphene citrate

The serum LH levels of different BMI groups during COH were presented in **Figure 2F**. In the basal state, the LH levels of group 2 and group 3 were lower than those of group 1. In the early follicular phase, the LH levels of all three groups gradually increased, with group 1 having the highest levels. Subsequently, the LH levels experienced a mild decrease due to negative feedback, which is consistent with the LH changes observed in the mild stimulation regimen of CC. On the trigger day and the day after the trigger day, the LH values of group 2 and group 3 were also significantly lower compared to group 1.

**Figure 3:**
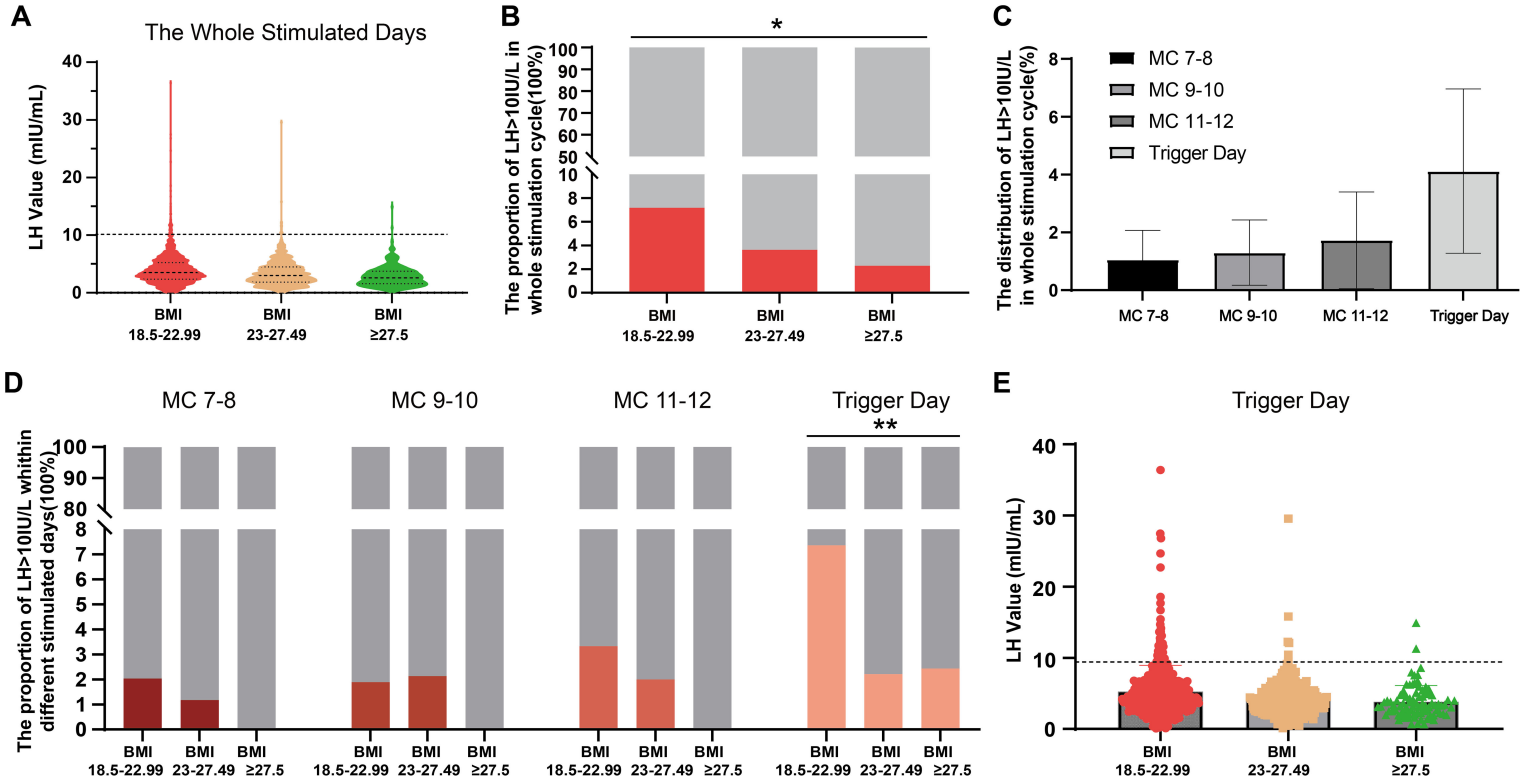
The proportion and distribution of LH >10 IU/L during controlled ovarian hyperstimulation with clomiphene citrate in women with normal and high BMI. **(A)** A violin plot is used to visualize the distribution of high LH values throughout the whole controlled ovarian hyperstimulation stage, excluding the day after trigger day. **(B)** The proportions of LH >10 IU/L are compared among three different BMI groups. **(C)** The distribution of LH>10 IU/L is shown at specific days during controlled ovarian hyperstimulation period with clomiphene citrate. **(D)** The detailed proportion of LH >10 IU/L is presented for each BMI group at specific days during controlled ovarian hyperstimulation with clomiphene citrate. **(E)** A scatterplot displays the LH values on the trigger day, which is when LH >10 IU/L most likely occurs. Categorical variables were expressed as proportions (%) and tested using chi-square test or Fisher’s test. *: p<0.05; **: p<0.01. LH, luteinizing hormone.

**Figure 3** displays the proportion and distribution of LH >10 IU/L among three BMI groups during COH. The violin plot in **Figure 3A** illustrates the distribution of high LH values across all days of COH (excluding the day after trigger day), providing a visual representation. The proportions of LH >10 IU/L for the three different BMI groups are illustrated in **Figure 3B**, with group 2 and group 3 exhibiting significantly smaller proportions compared to group 1 (7.19% vs 3.62% vs 2.27%, group 1-3, p<0.05). The distribution of LH>10 IU/L throughout the stimulation cycle, as depicted in **Figure 3C**, suggests a higher likelihood of its occurrence on the trigger day; however, this difference was not statistically significant (p=0.224). **Figure 3D** illustrates the precise distribution of LH >10 IU/L across different BMI groups during each COH period, aligning with the observed inverse relationship between BMI and the likelihood of LH >10 IU/L. The scatterplot in **Figure 3E** illustrates the distribution of LH values on the trigger day, which is the day with the highest likelihood of having an LH level greater than 10 IU/L. This analysis specifically focuses on data points where the LH value exceeds 10 IU/L.

### Pregnancy outcomes of women with normal and high BMI when using clomiphene citrate

The pregnancy outcomes resulting from frozen embryo transfer are presented in **Table 2**. **Figure 1** displays the number of women who did not receive viable embryos, did not undergo embryo transfer, and underwent fresh embryo transfers. The pregnancy outcomes were specifically analyzed for the first FET within this oocyte retrieval cycle among the three BMI groups. In group 1, 263 patients underwent FET; in group 2, 131 patients underwent FET and in group 3, 40 patients underwent FET. The endometrium preparation protocols were different among the three groups (p<0.01): hormone replacement therapy was most commonly used in group 1 (39.16%), natural cycles were most frequently used in group 2 (30.53%), and letrozole mild stimulation was most commonly adopted in group 3 (45.00%). Group 3 had greater endometrial thickness on the day of embryo transfer than groups 1 and 2. However, there was no significant difference between the different BMI groups in the average number of transferred embryos, implantation rate, biochemical pregnancy rate, clinical pregnancy rate, miscarriage rate, ectopic pregnancy rate, and live birth rate.

**Table 2:** Pregnancy outcomes of first frozen embryo transfer cycles in women with clomiphene by BMI category (kg/m^2^).

	Group 1 (BMI 18.5-22.99)	Group 2 (BMI 23-27.49)	Group 3 (BMI ≥27.5)	*P* value
**Number of FET cycles (n)**	263	131	40	
**Endometrium preparation protocol(n)**				**0.002**
Natural cycles (%)	52 (19.77%)	40 (30.53%)	5 (12.50%)	
Hormone replacement (%)	103 (39.16%)	35 (26.72%)	8 (20.00%)	
Letrozole mild stimulation (%)	56 (21.29%)	30 (22.90%)	18 (45.00%)	
HMG late stimulation (%)	52 (19.77%)	26 (19.85%)	9 (22.50%)	
**Endometrial thickness (mm)**	10.42±2.22^a^	11.02±2.90^b^	11.52±2.27^b^	**0.006**
**Average number of transferred embryos (n)**	1.76±0.43	1.74±0.44	1.85±0.36	0.361
Cleavage embryo(n)	1.60±0.71	1.56±0.72	1.70±0.61	0.568
Blastocyst(n)	0.16±0.434	0.18±0.47	0.15±0.53	0.930
**Implantation rate (%)**	32.11 (149/464)	34.21 (78/228)	31.08 (23/74)	0.820
**Biochemical pregnancy rate (%)**	54.75 (144/263)	59.54 (78/131)	55.00 (22/40)	0.657
**Clinical pregnancy rate (%)**	47.15 (124/263)	50.38 (66/131)	47.50 (19/40)	0.830
**Miscarriage rate (%)**	20.16 (25/124)	18.18 (12/66)	10.53 (2/19)	0.565
**Ectopic pregnancy (%)**	1.61 (2/124)	4.55 (3/66)	5.26 (1/19)	0.424
**Live birth rate (%)**	34.98 (92/263)	37.40 (49/137)	32.50 (13/34)	0.820

For continuous data, one-way ANOVA analysis was performed; for proportions, the chi-square test was utilized. *Post hoc* analyses were performed with the Bonferroni test. Different superscript letters indicate significant differences between groups. BMI, body mass index; FET, frozen embryo transfer; HMG, human menopausal gonadotropin. P < 0.05 was considered statistically significant.

Bolded words in the table denote the titles of the indicators, while bolded values signify statistical significance.

## Discussion

### Principal findings

Patients with obesity and normal menstrual cycles exhibited well-controlled LH levels when using CC for COH, demonstrating a significantly lower incidence of LH >10 IU/L compared to normal-weight patients. Moreover, they achieve comparable outcomes in terms of oocyte retrieval and embryo quality to normal-weight patients.

### Comparisons between the results of the current study and previous studies

Previous studies have demonstrated that the combined use of CC in patients with diminished ovarian reserve or unexplained infertility effectively suppresses the negative feedback of estrogen on the hypothalamus, stimulates pituitary gonadotrophins, enhances ovarian sensitivity, and ultimately leads to an improved number of oocytes and cumulative pregnancy rates. CC+HMG demonstrated a significantly high yield of oocytes and good-quality embryos, while utilizing lower doses of gonadotrophins per oocyte and embryo compared to the conventional gonadotrophin-releasing hormone (GnRH) antagonist protocol in patients with poor ovarian reserve (23). Low doses of CC can generate more oocytes with an optimal cumulative pregnancy rate and reduce gonadotropin dosage in women with unsuspected poor *in vitro* fertilization outcomes using the GnRH agonist protocol (24). However, due to its inherent mechanism as a selective estrogen receptor antagonist, the administration of CC in patients with normal ovarian reserve tends to induce excessive elevation of LH levels. Early studies have reported a 20 ± 25% probability of premature LH surge during COH (25, 26). Additionally, experiments conducted on rats have indicated that CC may directly act on the pituitary gland to facilitate estrogen-induced LH surge (27).

Former studies have indicated that obesity is characterized by poor hypothalamic-pituitary-ovarian responsiveness (5–7) and is associated with deficient secretion of LH, FSH, and sex steroids (8). A comprehensive epidemiological study involving 848 women revealed that overweight individuals with a body mass index of 25 kg/m^2^ exhibited longer follicular phases, lower levels of LH and FSH, as well as a 33% reduction in estrogen and progesterone metabolites compared to women with normal weight (28). Furthermore, obesity impairs the ovarian response to gonadotropin stimulation, leading to increased dosage and duration of gonadotropins, higher rates of cycle cancellation, and fewer retrieved oocytes (9, 10, 29, 30). Numerous previous studies have consistently demonstrated the detrimental impact of obesity on ovulation, embryo quality, and live birth rates (31); however, only a limited number of recent studies have shown that a COH program can mitigate these adverse effects associated with obesity. Roberto Marci et al. hypothesized that COH with GnRH antagonists is an effective and well-tolerated treatment option for women with high BMI compared to GnRH agonist protocols (32). John J. et al. discovered that obesity impacts oocyte quality in GnRH agonist long protocols, while BMI does not affect oocyte number and maturity in HMG+CC protocols, suggesting that mild ovarian stimulation may be more suitable for women with obesity (33).

In the existing literature (34–36), the majority of studies on obese patients treated with CC have primarily focused on comparing its efficacy with letrozole in inducing ovulation. Although some studies support the suitability of CC for patients with high BMI, particularly in mild ovarian stimulation protocols, there is a notable lack of research specifically investigating the incidence of premature LH surges in obese patients undergoing CC-assisted controlled ovarian hyperstimulation. Our study aims to address this significant gap by examining the occurrence of premature LH surges in obese women undergoing controlled ovarian hyperstimulation using CC. This study is unique in its consideration of the ability of clomiphene citrate to enhance LH levels and address the observed low LH levels in obese patients. Understanding these effects is crucial for assessing the safety and effectiveness of CC in this specific patient population.

Our study, for the first time, takes into account these mechanisms in previous research and proposes that clomiphene citrate may potentially compensate for the diminished hypothalamic-pituitary-ovarian responsiveness observed in women with obesity, thereby serving as a suitable option for controlled ovarian hyperstimulation treatment. We found that consistently lower LH levels were observed during COH with CC in patients with obesity compared to those with normal weight. This effective control of LH levels mitigates the occurrence of premature LH surge and reduces the need for frequent patient monitoring. More notably, our research revealed that the utilization of CC in patients with obesity and regular menstrual cycles exhibited comparable outcomes to those of normal weight patients regarding oocyte retrieval and embryo development. Our findings indicate that clomiphene citrate may effectively compensate for the poor pituitary responsiveness seen in patients with obesity and serve as a suitable option for controlled ovarian hyperstimulation treatment, thereby contributing to safer and more effective infertility management within this population.

### Clinical implications

The well-controlled LH levels during COH by CC, particularly the reduced occurrence of premature LH elevation in women with obesity and normal menstrual cycles, can be attributed to several potential mechanisms: [1] Impaired hypothalamic-pituitary responses during COH in individuals with obesity may result from obesity-induced insulin resistance, elevated levels of inflammatory factors and leptin, as well as decreased endogenous kisspeptin secretion (37, 38). [2] Conversely, CC can enhance FSH and LH levels by inhibiting estrogen’s negative feedback and improving pituitary sensitivity (27, 39), which proves advantageous for treating patients with obesity [3]; The improvement of pituitary sensitivity induced by CC can compensate for the reduced hypothalamic-pituitary function and LH levels observed in women with obesity. Consequently, the incidence of premature LH elevation in patients with obesity differs from that in normal-weight patients and is characterized by well-controlled LH levels.

The overall improvement in embryo and live birth outcomes in patients with obesity via CC may be attributed to the fact that well-controlled LH levels are more favorable for oocyte development and enhance oocyte developmental potential. In the context of COH, it is crucial to regulate the LH levels within an appropriate range. Elevated levels of LH may hinder the development of oocytes, resulting in granulosa cell luteinization and premature ovulation (40), which have been associated with decreased pregnancy rates and cumulative live birth rates, as indicated by certain retrospective studies (22, 41–43). Recent studies utilizing RNA sequencing have demonstrated that serum LH levels during COH influence the transcriptome profiles of ovarian granulosa cells (44). *In vitro* culture of ovarian granulosa cells exposed to excessive recombinant LH results in damage to mitochondria and other organelles. The latest review also suggests that excessive suppression of LH during ovarian stimulation may result in unfavorable outcomes in assisted reproduction (45), and the administration of recombinant LH could potentially benefit individuals with poor response rate (46). Therefore, it is possible that maintaining appropriate levels of LH contributes to why patients with obesity can achieve comparable outcomes to normal-weight patients with CC. Future research could delve deeper into these hypotheses and examine the impact of LH concentration on follicular growth.

### Strengths and limitations

In this study, we observed that patients with obesity undergoing COH with CC exhibited well-controlled LH levels compared to normal-weight patients, with a low incidence of LH exceeding 10 IU/L. The effective control of LH levels mitigates the occurrence of premature LH surge and eliminates the need for frequent patient monitoring. Furthermore, there were no discernible differences in oocyte retrieval, embryo, and pregnancy outcomes between patients with obesity and those with normal-weight. These findings suggest that HMG+CC represents an optimal COH protocol for patients with obesity and regular menstrual cycles.

The study is limited in several aspects. Firstly, its retrospective nature and single-center design may introduce bias, potentially compromising the representativeness and generalizability of research findings due to the exclusive use of samples from a specific medical institution. This bias can lead to selection bias, inadequate control of confounding factors, and challenges in result reproducibility, thereby impacting the accuracy and reliability of causal inference. Therefore, there is a need for multi-center prospective studies with larger and more diverse populations to further advance this research. Secondly, the sample size was relatively small, particularly for the third subgroup (BMI≥27.5) in the analysis of pregnancy outcomes. This may not provide sufficient statistical power to detect a significant impact on comparable pregnancy outcomes among obese patients undergoing FET. Therefore, further investigation with a larger cohort is warranted to confirm these findings. The potential confounding variables such as age distribution and pre-treatment protocols before CC could potentially influence our findings.

## Conclusion

The present study demonstrates that infertile patients with obesity and regular menstrual cycles have a low prevalence of premature LH elevation when administered CC as a COH regimen, and their outcomes in terms of oocyte retrieval and embryo development are comparable to those observed in normal-weight patients. Furthermore, pregnancy rates might be similar to those of normal-weight patients. These results suggest that the use of clomiphene citrate in obese women can effectively reduce premature LH elevation compared to those with normal weight and serve as a suitable option for controlled ovarian hyperstimulation treatment, thereby contributing to safer and more effective infertility management within this population.

## Data Availability

The raw data supporting the conclusions of this article will be made available by the authors, without undue reservation.
